# Effect of adding telerehabilitation home program to pharmaceutical treatment on the symptoms and the quality of life in children with functional constipation: a randomized controlled trial

**DOI:** 10.1007/s00431-024-05639-8

**Published:** 2024-06-26

**Authors:** Amir Soliman, Safy Eldin M. AboAli, Amel E. Abdel Karim, Sara A. Elsamahy, Judy Hasan, Badr Al-Amir Hassan, Amira H. Mohammed

**Affiliations:** 1 Department of Public Health and Community Medicine, Faculty of Medicine, Delta University for Science and Technology, Gamasa, Egypt; 2 Department of Physical Therapy for Pediatrics, Faculty of Physical Therapy, October University for Modern Sciences and Arts, Giza, Egypt; 3 Department of Physical Therapy for Pediatrics, Misr University for Science and Technology, Giza, Egypt; 4 Basic Science Department, Faculty of Physical Therapy, Misr University for Science and Technology, Giza, Egypt; 5 Dakahlia STEM School, Al-Mansoura, Egypt; 6 Department of Physical Therapy for Internal Medicine and Geriatrics, Faculty of Physical Therapy, Delta University for Science and Technology, Gamasa, Egypt; 7 Department of Physical Therapy for Pediatrics, Faculty of Physical Therapy, Delta University for Science and Technology, Gamasa, Egypt

**Keywords:** Physiotherapy, Conventional treatment, Functional constipation

## Abstract

Painful defecation, the passage of hard stools, unpleasant or irregular bowel deviation/movements from regular rate, and/or the feeling of not enough elimination of stool are common symptoms of functional constipation. The goals in treating constipation are to produce soft, painless stools and to prevent the re-accumulation of feces. This study looked at how the telerehabilitation home program (TRP) affected the symptoms of FC and the children who were constipated in terms of their quality of life. A randomized controlled trial included 400 children aging 4–18 years with functional constipation distributed in two groups: control group consisted of 200 children receiving pharmaceutical treatment and the intervention group consisted of 200 children receiving the telerehabilitation home program in addition to pharmaceutical treatment. Both groups received the interventions for 6 months. The outcomes in terms of functional constipation symptoms and quality of life are measured and compared pre- and post-interventions. Adding the telerehabilitation home program to pharmaceutical treatment of functional constipation in children results in prominent improvement in the condition; there is a significant difference between the intervention and control group in all Rome criteria which assess symptoms of functional constipation except rush to the bath-room to poop which showed non-significant difference; there is also a significant difference between the intervention and control group in all domains of the SF-36 questionnaire which assess quality of life except the mental health domain which showed non-significant difference.

*Conclusion*: Adding the telerehabilitation home program to pharmaceutical treatment of functional constipation in children results in prominent improvement in symptoms of functional constipation and quality of life.

*Trial registration*: Our study was registered retrospectively with Clinicaltrials.gov under the identifier NCT06207721 on 5 January 2024.

**Table:** 

**What Is Known:** *• Painful defecation, passage of hard stools, unpleasant or irregular bowel movements, feeling of incomplete stool elimination are common symptoms of functional constipation.* *• Goals in treating constipation include producing soft, painless stools and preventing re-accumulation of feces.* **What Is New:** *• Adding telerehabilitation home program to pharmaceutical treatment resulted in significant improvement in functional constipation symptoms and quality of life.* *• Significant differences between intervention group (200 children receiving telerehabilitation home program in addition to pharmaceutical treatment) and control group (200 children receiving pharmaceutical treatment) were observed in all Rome criteria except for rush to the bathroom to poop, and in all domains of SF36 questionnaire except for the mental health domain.*

**Supplementary Information:**

The online version contains supplementary material available at 10.1007/s00431-024-05639-8.

## Introduction

Constipation is a prevalent problem across all pediatric age groups, affecting 0.7 to 29.6% (average 12%) of children aged 0–18 years. Its severity varies from moderate to severe, and its duration can range from brief to chronic [1–4]. The Rome III criteria (Appendix I), developed in 2006 by pediatric gastroenterologists, include symptoms of constipation [3, 5]. The psychological functioning of children is significantly impacted by constipation. Roughly 40% of these children struggle with mental issues like depression, eating disorders, truancy, family-related matters, and a lack of socialization [6–10].

Childhood constipation is a complex and poorly understood etiology. More than 90% of children with constipation also referred to as functional constipation (FC) have no discernible biological reason. It is a typical pediatric issue, with a reported 3% frequency globally [4]. Dyssynergic defecation and delayed intestinal transit are two symptoms of functional constipation. Defecating incompletely due to paradoxical activation of the pelvic floor muscles or to not relaxing them during pushing and/or to not increasing intra-rectal stress is known as dyssynergic defecation, and it accounts for most juvenile difficulties [11, 12]. Dyssynergic defecation may be the cause of delayed intestinal transit in certain people. This irregular pattern of feces is seen in about 50% of children [13]. Most people agree that the etiology of dyssynergic constipation in children is either diligently uncomfortable defecation, fear of bowel movements, or retaining feces, which can lead to a continuous cycle [4, 7, 12, 14–16].

The primary objective of constipation treatment is to induce smooth, painless stools and to stop the re-accumulation of waste [17]. In addition to regular physical exercise and a pharmaceutical follow-up regimen after an extra pharmaceutical therapy for fecal disimpaction, the recommended care of FC involves an adequate dose of fibers and liquids [18].

Pelvic physiotherapists are expected to have a significant role in increasing the success rate, as dyssynergic dysfunction of the pelvic floor is the primary cause of FC in children. A typical regimen for pelvic physiotherapy includes breathing and relaxation exercises, toilet training, demystification, the use of micturition and defecation diaries, education, and pelvic floor muscle training, which includes exercises and biofeedback [19, 20]. It is still unclear how exercise and constipation are related, though, since some research suggests that moderate-intensity aerobic exercises might prevent constipation, while other research suggests that intense exercise, especially sudden changes in intensity, can exacerbate diarrhea, constipation, and stomach discomfort [21, 22].

The muscles around the lumbar spine, hips and pelvis, thoracolumbar fascia, and the spine and abdomen are considered core muscles. One method to relieve constipation and enhance gastrointestinal motility is to engage in core strengthening activities. The development of the diaphragm, multifidus, transverse abdominal, and pelvic floor muscles is also facilitated by specific core exercise training. By stimulating the gastrointestinal system, workouts that strengthen the core muscles raise intra-abdominal pressure, which may enhance colorectal motility [23]. Telerehabilitation (TR) is the utilization of telecommunications and electronic technological advances to support community health management, patient and medical practitioner learning, and distant practical rehabilitation. The objectives of TR are to conduct a distance-therapeutic intervention program, assess rehabilitative therapies and track their outcomes, and offer distance education and counselling. Research indicates that TR has potential applications in the field of pediatric rehabilitation and physiotherapy [24].

Despite the possible advantages, not much research has been done on how the quality of life (QL) and FC symptoms in children who are constipated are affected by the telerehabilitation home program (TRP). Thus, the current study looked at how the TRP affected the symptoms of FC and the children who were constipated in terms of their QL. We postulated that a 6-month TRP would lessen FC symptoms and enhance children’s QL when compared to a control group of children who did not exercise.

## Materials and methods

### Study design

This single-blinded randomized controlled study was carried out in compliance with the ethical principles of the 1975 Helsinki Declaration. It took place from October 2022 to December 2023. Our study was registered retrospectively with Clinicaltrials.gov under the identifier NCT06207721 on 5 January 2024.

Before conducting the study, the Faculty of Medicine, Al-Azhar University, Damietta, ethics committee board accepted our procedures (number, DFM-IRB000012367-24-03-024). All participants signed a consent form after hearing an explanation of the procedure. They are aware that they can withdraw their consent and quit taking part in the study at any time without harming the researchers.

### Participants

Four hundred children diagnosed with functional constipation were randomly selected to participate in the study. All subjects were recruited from general practitioner and pediatric outpatient clinic in general and central hospitals in Damietta governorate, Egypt. They were selected according to the subsequent criteria: The participants ranged in age from 4 to 18 years old, from both sex, diagnosed to have FC according to Rome criteria III (appendix I) [3, 5]. They had normal or healthy weight according to Centers for Disease Control and Prevention (CDC) growth charts for girls which ranged from body mass index (BMI) for age 85th to 95th percentiles [25].

Children with severe delay in motor skills development, constipation caused by medicine, bowel surgery (except appendectomy), endocrine and metabolic disorders (diabetes mellitus, hypercalcemia, hypothyroidism, and diabetes insipidus), psychiatric and neurological disorders (cerebral palsy, spina bifida, PDD-NOS, autism, or anorexia nervosa), Hirschsprung’s disease, or Down syndrome were excluded from this study.

### Randomization and blinding

Four hundred and twenty-five children diagnosed with functional constipation were assessed for suitability; due to their failure to fulfil the inclusive criteria, 20 participants were eliminated from the experiment, and five parents refused their children to join. As a result, 400 children diagnosed with FC were involved in this experimental. Random allocation software was used to split them into two equal-sized groups at random in order to minimize selection bias [26]. The outcome assessor was blind to recruitment, randomization, and allocation of subjects.

**Fig. 1:**
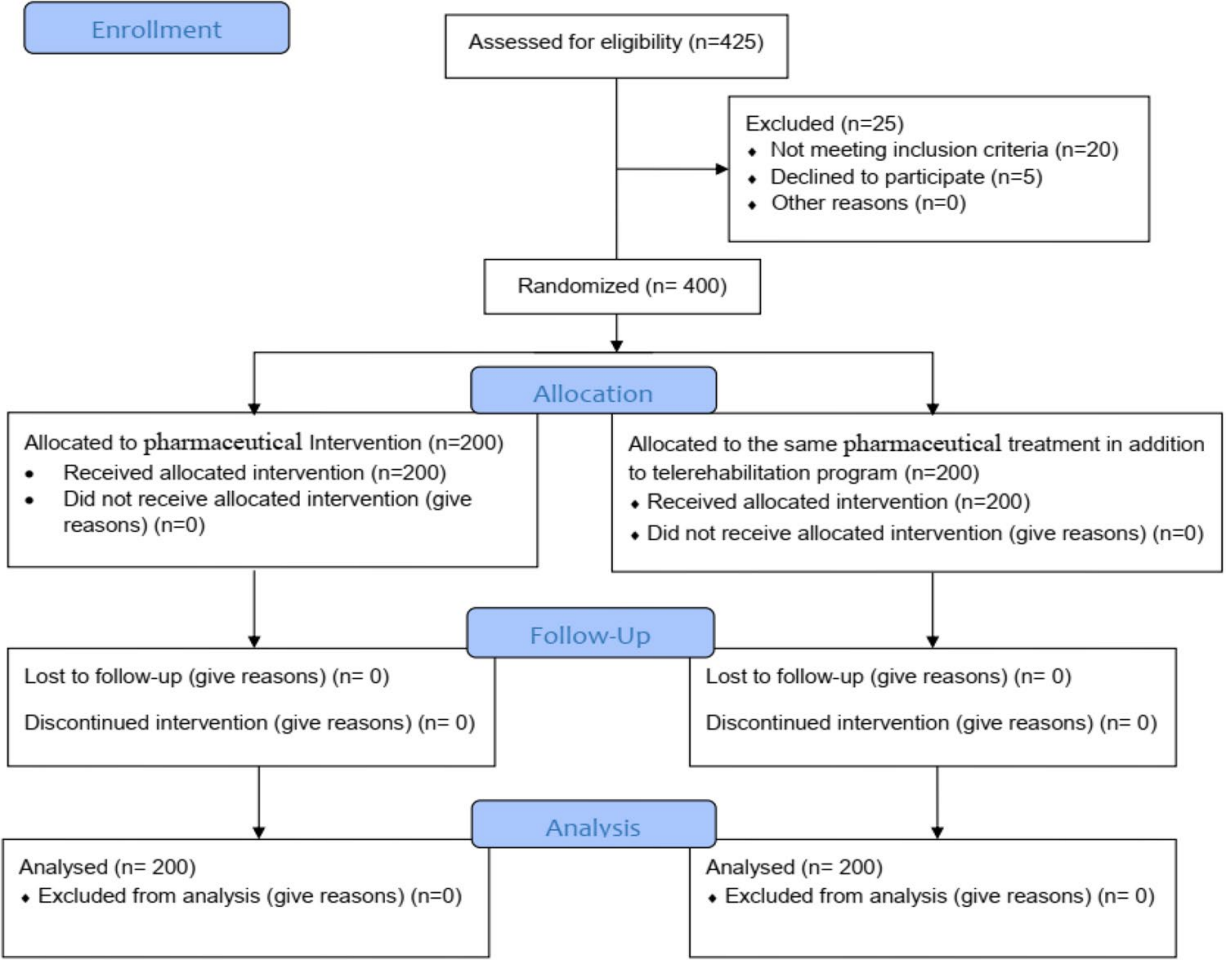
CONSORT flow chart

The children were sorted into two equal groups at random through the GraphPad QuickCalcs website [27] (*n* = 200 in each group): group A (control group), which treated with pharmaceutical treatment (including diet regimen, and laxatives as polyethylene glycol (PEG) syrup (0.7 g/kg daily); and group B (intervention group), which treated with the same pharmaceutical treatment conducted to group A in adding to TRP. A depiction of participant retention and randomization during the study is presented in Fig. 1.

### Outcome measures

All participants were examined at the start of the treatment and again 6 months later. They were assessed for the following outcomes:

- The primary outcome: Rome III Diagnostic Questionnaire for the Pediatric Functional Gastrointestinal Disorders (appendix I) [3, 5]

It was used as an indicator for the treatment success which is defined according to the Rome III criteria as higher improvement of FC symptoms with less or no laxative use.

- The secondary outcome: Short form 36 (SF-36) questionnaire (appendix II)

The SF-36 scale for evaluating the QL was determined to be credible and accurate and thought to be acceptable for Arab populations, particularly in North Africa, after having been adapted and altered in Tunisia and utilized in research on Tunisian participants [28]. The Arabic version proved to be an objective, simple, and legitimate instrument for measuring the health-associated QL in the general population as a whole [29].

The Arabic-translated short form 36 questionnaire is a widely used self-administered measure for evaluating the QL. It has 36 questions. The results are converted into a scale of 0 to 100, with higher values indicating better QL. The questionnaire was divided into eight domains: physical functioning, general health, bodily pain, role functioning, social functioning, role emotional, mental health, and vitality. Our team contacted general practitioner or pediatrician outpatients’ clinics in general and central hospitals in Damietta governorate to explain the objectives of the study. Letters revealing the goals of the research were given to the families, which included an interview during which two members of the team responded to the queries of the parent(s)/guardian(s). If the invitation to join the research was accepted, a written agreement was agreed upon, then the questionnaire forms were filled at the start of the study and after 6 months of follow-up by participant children aging 12 years and more, and by parents of participant children aging below 12 years, in both situations the questionnaire forms were submitted under the guidance of a member of the team with the utmost level of privacy [29].

### Interventions

Two groups received the following treatment: group A (control group), which received pharmaceutical treatment (including diet regimen, and laxatives as polyethylene glycol (PEG) syrup (0.7 g/kg daily); and group B (intervention group), which received the same pharmaceutical treatment conducted to group A in addition to TRP. Our team contacted general practitioners or pediatrician outpatients’ clinics in general and central hospitals in Damietta governorate to monitor with the children’s families the diet regimen, the dose of laxatives, and any adverse effects of laxatives, e.g., abdominal pain, vomiting, nausea, and abnormalities [30].

The telerehabilitation home program was conducted at home by parent(s)/guardian(s) who were trained and supervised by a physiotherapist. The telerehabilitation home program included the following: (i) isometric training of the abdominal muscles, (ii) breathing exercises, and (iii) abdominal massage.

The target of isometric training of the abdominal muscles is to rise intra-abdominal pressure (that compresses the bowels) and the propulsive force of the colon throughout intentional power [31, 32]. Bearing in mind the secondary synergic stimulation stuck between the muscles of pelvic floor and the muscles of lower abdomen, intentional isometric contraction of upper abdomen with coincident relaxation of lower abdomen increases coordination of muscles and relaxation of the external anal sphincter and pelvic floor, so improving defecation [31, 33, 34]. The exercises were performed in while the participant was either in the position of lying down in left lateral decubitus position with flexed knee and hip at right angle or in the position of sitting. In the first position, the exercise is initiated with two sequences of 8 contractions and relaxations for 3 months, then upgraded to two sequences of 12 contractions and relaxations for the following 3 months. In the second position (sitting), the exercise is initiated with one sequence of 3 contractions and relaxations taking 10 s for 3 months then upgraded to five sequences for the following 3 months. Success of the exercise is considered when the lower abdominal protrusion is obvious, which represents the concurrent relaxation of lower abdomen and pelvic floor, and the series starts from this point [34]. The goal of breathing exercises is to reach a steady form of abdominal breathing, fortify the muscles of abdomen, and increase harmonization between breathing, contraction of abdominal and anal muscles, and propulsions of colon. Standard diaphragmatic breathing was attained via an adjusted exercise while with the participant in the sit down position, one hand is located over the abdomen, and the other hand over the thorax; the participant is ordered to breathe in little by little, profoundly and increasingly for 6 to 8 s, with air retention for 10 s and thin breathe out slowly for 6 to 8 s. Two sequences of ten repetitions are accomplished. Success of the exercise is considered when superior motion of the hand placed over the abdomen is reached in comparison with less or no movement of the hand over the thorax, and the series starts from this point [35]. The goal of abdominal massage is to achieve propulsive massage of the abdomen to enhance motility of colon and rectum to improve intestinal functions and defecation [14, 36, 37]. Sluggish rounded clockwise movements are done, along the contour of the colon, with continuous adequate pressure on the abdomen using a steady tennis ball on every point for 1 min, starting by the ascending colon and moving on the way to the sigmoid colon.

No noticeable detrimental effects were seen while the treatments were in use. Each participant was asked to explain any concerns they were experiencing.

### Power analysis

The sample size was estimated using the OpenEpi I program with a 95% confidence level and 80% power. We intended to have the ability to find a variance of no less than 15% in the result between the two groups; thus, we required a minimum of 200 participants in each group.

### Statistical analysis

Data from history, clinical examination, laboratory tests, and outcome measures were input and analyzed using Microsoft Excel. Data was inputted into the computer and analyzed with IBM SPSS software version 26 (Armonk, NY, IBM Corp). The qualitative findings have been explained using numbers and percentages. The normality of the distribution was verified using the Shapiro-Wilk test. The Student *T* test was performed to determine the statistically significant value of the variance between two study groups’ averages. The Mann-Whitney *U* test is a nonparametric test that allows two groups or conditions or treatments to be compared without making an assumption that values are normally distributed. The chi-square test was used to examine the relationship between two or more qualitative variables. The Monte Carlo test was used to investigate the association between two groups with qualitative factors when the predicted count exceeds 5 in more than 20% of cells. Results were classified as non-significant (*P* > 0.05), significant (*P* < 0.05), or highly significant (*P* < 0.001). The data related with the study are not publicly available; however, they are available from the corresponding author upon reasonable request.

## Results

### Demographic data

Table 1 shows the characteristics of children, including age and gender. The features of the children in both groups were not substantially different (*P* > 0.05).

**Table 1:** Comparison between age and gender of participants in TRP group and control group

**Variables**	**Control group** **(*n*** **= 200)**	**TRP group** **(*n*** **= 200)**	**Test of significance**
**Age (year)**			
**Mean ± SD**	11.12 ± 3.86	10.75 ± 3.67	*t* = 0.96 *P* = 0.33
**Gender**			
**Male**	95	85	*χ* = 1.01 *P* = 0.31
**Female**	105	115	

Data are expressed as mean ± SD

*t*, independent samples *t*-test; *χ*, chi-square test

*Statistically significant (*P* < 0.05)

### The primary outcome: Rome III Diagnostic Questionnaire for the Pediatric Functional GI Disorders

Comparing the pre- and post-treatment mean values of the items of Rome III Diagnostic Questionnaire for the Pediatric Functional GI Disorders in the control group represented significant difference as *P* < 0.001 in all items except the 2a, 3rd, 9th, and 10th items as *P* = 0.009, *P* = 1, *P* = 0.33, and *P* = 0.21 respectively (Table 2). Comparing the pre- and post-treatment mean values of the items of Rome III Diagnostic Questionnaire for the Pediatric Functional GI Disorders in the TRP group represented significant difference in all items as *P* < 0.001 (Table 3). Comparing the post-treatment mean values of the items of Rome III Diagnostic Questionnaire for the Pediatric Functional GI Disorders in both groups showed high statistically significant difference between both groups as *P* < 0.001 except the 4th items regarding the rush to the bath-room to poop as *P* = 0.314 (Table 4).

**Table 2:** Analysis of Rome III Diagnostic Questionnaire for the Pediatric Functional GI Disorders in the control group pre- and post-pharmaceutical treatment

**Variables**	**Pre-treatment** **(*n*** **= 200)**	**Post-treatment** **(*n*** **= 200)**	**Test of significance**
1. In the last 2 months, how often did you usually have poops?			
▪ 2 times a week or less often	180	160	MC = 14.51 *P* = 0.001*
▪ 3 to 6 times a week	20	28	
▪ Once a day	0 (0%)	12	
▪ 2 to 3 times a day	0 (0%)	0 (0%)	
▪ More than 3 times a day	0 (0%)	0 (0%)	
2. In the last 2 months, what was your poop usually like?			
▪ Very hard	132	116	MC = 35.166 *P* < 0.001*
▪ Hard	68	52	
▪ Not too hard and not too soft	0 (0%)	16	
▪ Very soft or mushy	0 (0%)	0 (0%)	
▪ Watery	0 (0%)	0 (0%)	
▪ It depends (my poops are not always the same)	0 (0%)	16 (8%)	
2a. If your poops were usually hard, for how long have they been hard?			
▪ Less than 1 month	0 (0%)	8	MC = 9.40 *P* = 0.009
▪ 1 month	112	92	
▪ 2 months	88	84	
▪ 3 or more months	0 (0%)	0 (0%)	
3. In the last 2 months, did it hurt when you had a poop?			
▪ No	80	80	*χ*^2^ = 0 *P* = 1
▪ Yes	120	120	
4. Did you have to rush to the bath-room to poop?			
▪ Never	0 (0%)	52	MC = 213.07 *P* < 0.001*
▪ Once in a while	0 (0%)	84	
▪ Sometimes	20	16	
▪ Most of the time	112	32	
▪ Always	68	16	
5. Did you have to strain (push hard) to make a poop come out?			
▪ Never	0 (0%)	4	MC = 98.11 *P* < 0.001*
▪ Once in a while	0 (0%)	72 (36%)	
▪ Sometimes	20	20 (10%)	
▪ Most of the time	112	56	
▪ Always	68	48	
6. Did you pass mucus or phlegm (white, yellowish, stringy, or slimy material) during a poop?			
▪ Never	0 (0%)	20 (10%)	MC = 221.03 *P* < 0.001*
▪ Once in a while	0 (0%)	112	
▪ Sometimes	20 (10%)	24 (12%)	
▪ Most of the time	112	16 (8%)	
▪ Always	68	28 (14%)	
7. Did you have a feeling of not being finished after a poop (like there was more that wouldn’t come out)?			
▪ Never	0 (0%)	8 (4%)	MC = 103.81 *P* < 0.001*
▪ Once in a while	0 (0%)	72 (36%)	
▪ Sometimes	20 (10%)	20 (10%)	
▪ Most of the time	112	56 (28%)	
▪ Always	68	44 (22%)	
8. In the last 2 months, did you have a poop that was so big that it clogged the toilet?			
▪ No	16 (8%)	60 (30%)	*χ*^2^ = 31.44 *P* < 0.001*
▪ Yes	184 (92%)	140 (70%)	
9. Some children hold in their poop even when there is a toilet they could use. They may do this by stiffening their bodies or crossing their legs. In the last 2 months, while at home, how often did you try to hold in a poop?			
▪ Never	36 (18%)	36 (18%)	MC = 4.57 *P* = 0.33
▪ 1 to 3 times a month	68 (34%)	68 (34%)	
▪ Once a week	48 (24%)	36 (18%)	
▪ Several times a week	40 (20%)	44 (22%)	
▪ Every day	8 (4%)	16 (8%)	
10. Did a doctor or nurse ever examine you and say that you had a huge poop inside?			
▪ No	52 (26%)	60 (30%)	*χ*^2^ = 0.794 *P* = 0.21
▪ Yes	148 (74%)	140 (70%)	
11. In the last 2 months, how often was your underwear stained or soiled with poop?			
▪ Never	0 (0%)	16 (8%)	MC = 164.40 *P* < 0.001*
▪ Less than once a month	4 (2%)	48 (24%)	
▪ 1 to 3 times a month	12 (6%)	64 (32%)	
▪ Once a week	56 (28%)	44 (22%)	
▪ Several times a week	76 (38%)	28 (14%)	
▪ Every day	52 (26%)	0 (0%)	
11. a. When you stained or soiled underwear, how much was it stained or soiled?			
▪ Underwear was stained (no poop)	76 (38%)	32 (16.9%)	MC = 42.56 *P* < 0.001*
▪ Small amount of poop in underwear (less than a whole poop)	80 (40%)	76 (38%)	
▪ Large amount of poop in underwear (a whole poop)	44 (22%)	76 (38%)	
11. b. For how long have you stained or soiled your underwear?			
▪ 1 month or less	4 (2%)	12 (6%)	MC = 54.09 *P* < 0.001*
▪ 2 months	52 (26%)	60 (30%)	
▪ 3 months	80 (40%)	88 (44%)	
▪ 4 to 11 months	32 (16%)	24 (12%)	
▪ 1 year or longer	32 (16%)	0 (0%)	

Data are expressed as number (percent)

*MC*, Monte Carlo test; *χ*^*2*^, chi-square test

*Statistically significant (*P* < 0.05)

**Table 3:** Analysis of Rome III Diagnostic Questionnaire for the Pediatric Functional GI Disorders in the TRP group pre- and post-intervention

**Variables**	**Pre-treatment** **(*n*** **= 200)**	**Post-treatment** **(*n*** **= 200)**	**Test of significance**
1. In the last 2 months, how often did you usually have poops?			
▪ 2 times a week or less often	164 (83%)	84 (42%)	MC = 74.30 *P* < 0.001*
▪ 3 to 6 times a week	36 (18%)	92 (46%)	
▪ Once a day	0 (0%)	24 (12%)	
▪ 2 to 3 times a day	0 (0%)	0 (0%)	
▪ More than 3 times a day	0 (0%)	0 (0%)	
2. In the last 2 months, what was your poop usually like?			
▪ Very hard	144 (72%)	16 (8%)	MC = 222.933 *P* < 0.001*
▪ Hard	56 (28%)	64 (32%)	
▪ Not too hard and not too soft	0	100 (50%)	
▪ Very soft or mushy	0 (0%)	0 (0%)	
▪ Watery	0 (0%)	0 (0%)	
▪ It depends (my poops are not always the same)	20 (10%)	16 (8%)	
2a. If your poops were usually hard, for how long have they been hard?			
▪ Less than 1 month	4 (2%)	16 (8%)	MC = 32.76 *P* < 0.001*
▪ 1 month	8 (4%)	0 (0%)	
▪ 2 months	80 (40%)	20 (10%)	
▪ 3 or more months	108 (54%)	64 (32%)	
3. In the last 2 months, did it hurt when you had a poop?			
▪ No	32 (16%)	164 (82%)	*χ*^2^ = 174.31 *P* < 0.001*
▪ Yes	168 (84%)	36 (18%)	
4. Did you have to rush to the bath-room to poop?			
▪ Never	0 (0%)	56 (28%)	MC = 151.61 *P* < 0.001*
▪ Once in a while	0 (0%)	48 (24%)	
▪ Sometimes	16 (8%)	20 (10%)	
▪ Most of the time	76 (38%)	40 (20%)	
▪ Always	108 (54%)	36 (18%)	
5. Did you have to strain (push hard) to make a poop come out?			
▪ Never	0 (0%)	128 (64%)	MC = 400 *P* < 0.001*
▪ Once in a while	0 (0%)	72 (36%)	
▪ Sometimes	16 (8%)	0 (0%)	
▪ Most of the time	76 (38%)	0 (0%)	
▪ Always	108 (54%)	0 (0%)	
6. Did you pass mucus or phlegm (white, yellowish, stringy, or slimy material) during a poop?			
▪ Never	0 (0%)	88 (44%)	MC = 349.24 *P* < 0.001*
▪ Once in a while	0 (0%)	88 (44%)	
▪ Sometimes	16 (8%)	20 (10%)	
▪ Most of the time	76 (38%)	4 (2%)	
▪ Always	108 (54%)	0 (0%)	
7. Did you have a feeling of not being finished after a poop (like there was more that wouldn’t come out)?			
▪ Never	0 (0%)	108 (54%)	MC = 334.28 *P* < 0.001*
▪ Once in a while	0 (0%)	72 (36%)	
▪ Sometimes	16 (8%)	8 (4%)	
▪ Most of the time	76 (38%)	8 (4%)	
▪ Always	108 (54%)	4 (2%)	
8. In the last 2 months, did you have a poop that was so big that it clogged the toilet?			
▪ No	20 (10%)	128 (64%)	*χ*^2^ = 125.09 *P* < 0.001*
▪ Yes	180 (90%)	72 (36%)	
9. Some children hold in their poop even when there is a toilet they could use. They may do this by stiffening their bodies or crossing their legs. In the last 2 months, while at home, how often did you try to hold in a poop?			
▪ Never	108 (54%)	108 (54%)	MC = 12 *P* < 0.001*
▪ 1 to 3 times a month	40 (20%)	40 (20%)	
▪ Once a week	40 (20%)	24 (12%)	
▪ Several times a week	8 (4%)	12 (6%)	
▪ Every day	4 (2%)	16 (8%)	
10. Did a doctor or nurse ever examine you and say that you had a huge poop inside?			
▪ No	48 (24%)	128 (64%)	*χ*^2^ = 64.93 *P* < 0.001*
▪ Yes	152 (76%)	72 (36%)	
11. In the last 2 months, how often was your underwear stained or soiled with poop?			
▪ Never	0 (0%)	156 (78%)	MC = 389.33 *P* < 0.001*
▪ Less than once a month	0 (0%)	40 (20%)	
▪ 1 to 3 times a month	8 (4%)	4 (2%)	
▪ Once a week	68 (34%)	0 (0%)	
▪ Several times a week	72 (36%)	0 (0%)	
▪ Every day	52 (26%)	0 (0%)	
11. a. When you stained or soiled underwear, how much was it stained or soiled?			
▪ Underwear was stained (no poop)	56 (28%)	40 (90.9%)	MC = 291.46 *P* < 0.001*
▪ Small amount of poop in underwear (less than a whole poop)	76 (38%)	4 (9.1%)	
▪ Large amount of poop in underwear (a whole poop)	68 (34%)	0 (0%)	
11. b. For how long have you stained or soiled your underwear?			
▪ 1 month or less	16 (8%)	36 (81.8%)	MC = 332.835 *P* < 0.001*
▪ 2 months	20 (10%)	8 (18.2%)	
▪ 3 months	40 (20%)	0 (0%)	
▪ 4 to 11 months	68 (34%)	0 (0%)	
▪ 1 year or longer	56 (28%)	0 (0%)	

Data are expressed as number (percent)

*MC*, Monte Carlo test; *χ*^*2*^, chi-square test

*Statistically significant (*P* < 0.05)

**Table 4:** Analysis of Rome III Diagnostic Questionnaire for the Pediatric Functional GI Disorders in both groups post-treatment

**Variables**	**TRP group** **(*n*** **= 200)**	**Control group** **(*n*** **= 200)**	**Test of significance**
1. In the last 2 months, how often did you usually have poops?			
▪ 2 times a week or less often	84 (42%)	160 (80%)	MC = 15.461 *P* < 0.001*
▪ 3 to 6 times a week	92 (46%)	28 (14%)	
▪ Once a day	24 (12%)	12 (6%)	
▪ 2 to 3 times a day	0 (0%)	0 (0%)	
▪ More than 3 times a day	0 (0%)	0 (0%)	
2. In the last 2 months, what was your poop usually like?			
▪ Very hard	16 (8%)	116 (58%)	MC = 34.568 *P* < 0.001*
▪ Hard	64 (32%)	52 (26%)	
▪ Not too hard and not too soft	100 (50%)	16 (8%)	
▪ Very soft or mushy	0 (0%)	0 (0%)	
▪ Watery	0 (0%)	0 (0%)	
▪ It depends (my poops are not always the same)	20 (10%)	16 (8%)	
2a. If your poops were usually hard, for how long have they been hard?			
▪ Less than 1 month	16 (16%)	8 (4.3%)	MC = 7.345 *P* = 0.025*
▪ 1 month	0 (0%)	0 (0%)	
▪ 2 months	20 (20%)	92 (50%)	
▪ 3 or more months	64 (64%)	84 (45.7%)	
3. In the last 2 months, did it hurt when you had a poop?			
▪ No	164 (82%)	80 (40%)	*χ*^2^ = 18.537 *P* < 0.001*
▪ Yes	36 (18%)	120 (60%)	
4. Did you have to rush to the bath-room to poop?			
▪ Never	56 (28%)	52 (26%)	MC = 4.748 *P* = 0.314
▪ Once in a while	48 (24%)	84 (42%)	
▪ Sometimes	20 (10%)	16 (8%)	
▪ Most of the time	40 (20%)	32 (16%)	
▪ Always	36 (18%)	16 (8%)	
5. Did you have to strain (push hard) to make a poop come out?			
▪ Never	128 (64%)	4 (2%)	MC = 60.121 *P* < 0.001*
▪ Once in a while	72 (36%)	72 (36%)	
▪ Sometimes	0 (0%)	20 (10%)	
▪ Most of the time	0 (0%)	56 (28%)	
▪ Always	0 (0%)	48 (24%)	
6. Did you pass mucus or phlegm (white, yellowish, stringy, or slimy material) during a poop?			
▪ Never	88 (44%)	20 (10%)	MC = 20.315 *P* < 0.001*
▪ Once in a while	88 (44%)	112 (56%)	
▪ Sometimes	20 (10%)	24 (12%)	
▪ Most of the time	4 (2%)	16 (8%)	
▪ Always	0 (0%)	28 (14%)	
7. Did you have a feeling of not being finished after a poop (like there was more that wouldn’t come out)?			
▪ Never	108 (54%)	8 (4%)	MC = 40.171 *P* < 0.001*
▪ Once in a while	72 (36%)	72 (36%)	
▪ Sometimes	8 (4%)	20 (10%)	
▪ Most of the time	8 (4%)	56 (28%)	
▪ Always	4 (2%)	44 (22%)	
8. In the last 2 months, did you have a poop that was so big that it clogged the toilet?			
▪ No	128 (64%)	60 (30%)	*χ*^2^ = 11.602 *P* = 0.001*
▪ Yes	72 (36%)	140 (70%)	
9. Some children hold in their poop even when there is a toilet they could use. They may do this by stiffening their bodies or crossing their legs. In the last 2 months, while at home, how often did you try to hold in a poop?			
▪ Never	108 (54%)	36 (18%)	MC = 15.986 *P* = 0.003*
▪ 1 to 3 times a month	40 (20%)	68 (34%)	
▪ Once a week	24 (12%)	36 (18%)	
▪ Several times a week	12 (6%)	44 (22%)	
▪ Every day	16 (8%)	16 (8%)	
10. Did a doctor or nurse ever examine you and say that you had a huge poop inside?			
▪ No	128 (64%)	60 (30%)	*χ*^2^ = 11.602 *P* = 0.001*
▪ Yes	72 (36%)	140 (70%)	
11. In the last 2 months, how often was your underwear stained or soiled with poop?			
▪ Never	156 (78%)	16 (8%)	MC = 59.905 *P* < 0.001*
▪ Less than once a month	40 (20%)	48 (24%)	
▪ 1 to 3 times a month	4 (2%)	64 (32%)	
▪ Once a week	0 (0%)	44 (22%)	
▪ Several times a week	0 (0%)	28 (14%)	
▪ Every day	0 (0%)	0 (0%)	
11. a When you stained or soiled underwear, how much was it stained or soiled?			
▪ Underwear was stained (no poop)	40 (90.9%)	32 (17.4%)	MC = 22.632 *P* < 0.001*
▪ Small amount of poop in underwear (less than a whole poop)	4 (9.1%)	76 (41.3%)	
▪ Large amount of poop in underwear (a whole poop)	0 (0%)	76 (41.3%)	
11. b For how long have you stained or soiled your underwear?			
▪ 1 month or less	36 (81.8%)	12 (6.5%)	MC = 31.222 *P* < 0.001*
▪ 2 months	8 (18.2%)	60 (32.6%)	
▪ 3 months	0 (0%)	88 (47.8%)	
▪ 4 to 11 months	0 (0%)	24 (13%)	
▪ 1 year or longer	0 (0%)	0 (0%)	

Data are expressed as number (percent)

*MC*, Monte Carlo test; *χ*^*2*^, chi-square test

*Statistically significant (*P* < 0.05)

### The secondary outcome: domains of 36-Item Short Form Survey Instrument (SF-36)

Comparing the pre- and post-treatment mean values of the domains of SF-36 in the control group represented significant difference as *P* < 0.001 in all domains except the physical functioning as *P* = 0.009 (Table 5). Comparing the pre- and post-treatment mean values of the domains of SF-36 in the TRP group represented significant difference in all domains as *P* < 0.001 (Table 6). Comparing the post-treatment mean values of the domains of SF-36 in both groups showed high statistically significant difference between both groups as *P* < 0.001 (Table 7).

**Table 5:** Analysis of 36-Item Short Form Survey Instrument (SF-36) in the control group pre- and post-pharmaceutical treatment

**Variables**	**Pre-treatment** **(*n*** **= 200)**	**Post-treatment** **(*n*** **= 200)**	**Test of significance**
**Physical functioning**			
**Mean ± SD**	16.20 ± 16.73	21.60 ± 21.79	*z* = −2.61 *P* = 0.009
**Range**	0–50	0–60	
**Role of physical functioning**			
**Mean ± SD**	9.50 ± 14.07	17.50 ± 17.54	*z* = −4.78 *P* < 0.001*
**Range**	0–50	0–50	
**Role emotional**			
**Mean ± SD**	5.33 ± 13.95	16.66 ± 25.22	*z* = −5.26 *P* < 0.001*
**Range**	0–66.6	0–100	
**Bodily pain**			
**Mean ± SD**	42.75 ± 14.72	56.70 ± 16.17	*t* = 9.01 *P* < 0.001*
**Range**	45–100	22.5–87.5	
**General health**			
**Mean ± SD**	30.44 ± 9.91	38.64 ± 10.35	*t* = 8.08 *P* < 0.001*
**Range**	40–100	1–60	
**Vitality**			
**Range**	50	50	
**Social functioning**			
**Mean ± SD**	27.400 ± 16.40	39.05 ± 18.04	*z* = −6.67 *P* < 0.001*
**Range**	0–75	2.5–87.5	
**Mental health**			
**Mean ± SD**	38.40 ± 6.00	46.96 ± 4.52	*t* = 16.10 *P* < 0.001*
**Range**	40–60	40–60	
**Physical health dimension**			
**Mean ± SD**	117.06 ± 36,28	158.52 ± 41.86	*t* = 10.58 *P* < 0.001*
**Range**	172–442	89–225	
**Mental health dimension**			
**Mean ± SD**	112.52 ± 17.13	151.22 ± 19.67	*t* =20.97 *P* < 0.001*
**Range**	203–291	110–213	
**Physical mental aspect dimension**			
**Mean ± SD**	573.95 ± 114.63	774.35 ± 118.45	*t* = 17.19 *P* < 0.001*
**Range**	940–1820	557–982	
**Total score**			
**Mean ± SD**	32.91 ± 6.81	44.04 ± 6.87	*t* = 16.27 *P* < 0.001*
**Range**	55–103.89	32.92–56.67	

Data are expressed as mean ± SD / median (range)

*t*, independent samples *t*-test; *z*, Mann-Whitney *U* test

*Statistically significant (*P* < 0.05)

**Table 6:** Analysis of 36-Item Short Form Survey Instrument (SF-36) in the TRP group pre- and post-intervention

**Variables**	**Pre-treatment** **(*n*** **= 200)**	**Post-treatment** **(*n*** **= 200)**	**Test of significance**
**Physical functioning**			
**Mean ± SD**	22.212 ± 15.42	59.80 ± 31.46	*z* = −13.07 *P* < 0.001*
**Range**	0–100	0–60	
**Role of physical functioning**			
**Mean ± SD**	12.00 ± 20.20	77.50 ± 20.98	*z* = −16.77 *P* < 0.001*
**Range**	50–100	0–50	
**Role emotional**			
**Mean ± SD**	6.66 ± 13.36	90 ± 16.70	*z* = −18.306 *P* < 0.001*
**Range**	33.33–100	0–100	
**Bodily pain**			
**Mean ± SD**	31.300 ± 12.0743	74.400 ± 13.1531	*t* = 34.138 *P* < 0.001*
**Range**	45–100	22.5–87.5	
**General health**			
**Mean ± SD**	42.500 ± 11.7019	66.90 ± 14.93	*t* = 18.188 *P* < 0.001*
**Range**	40–100	1–60	
**Vitality**			
**Range**	50	50	
**Social functioning**			
**Mean ± SD**	33.000 ± 15.1955	66.75 ± 16.92	*z* = −14.613 *P* < 0.001*
**Range**	25–100	2.5–87.5	
**Mental health**			
**Mean ± SD**	35.920 ± 7.9593	48.08 ± 5.16	*t* = 18.123 *P* < 0.001*
**Range**	40–60	40–60	
**Physical health dimension**			
**Mean ± SD**	135.924 ± 35.5722	318.26 ± 70.40	*t* = 32.690 *P* < 0.001*
**Range**	172–442	89–225	
**Mental health dimension**			
**Mean ± SD**	122.500 ± 22.1364	235.68 ± 23.03	*t* =50.096 *P* < 0.001*
**Range**	203–291	110–213	
**Physical mental aspect dimension**			
**Mean ± SD**	646.06 ± 124.50	1384.85 ± 213.66	*t* = 42.250 *P* < 0.001*
**Range**	940–1820	557–982	
**Total score**			
**Mean ± SD**	38.42 ± 6.90	79.46 ± 11.86	*t* = 42.285 *P* < 0.001*
**Range**	55–103.89	32.92–56.67	

Data are expressed as mean ± SD / median (range)

*t*, independent samples *t*-test; *z*, Mann-Whitney *U* test

*Statistically significant (*P* < 0.05)

**Table 7:** Analysis of 36-Item Short Form Survey Instrument (SF-36) in both groups post-treatment.

**Variables**	**Control group** **(*n*** **= 200)**	**TRP group** **(*n*** **= 200)**	**Test of significance**
**Physical functioning**			
**Mean ± SD**	59.80 ± 31.70	21.60 ± 21.96	*z* = −5.989 *P* < 0.001*
**Range**	0–100	0–60	
**Role of physical functioning**			
**Mean ± SD**	77.50 ± 20.98	17.50 ± 17.68	*z* = −8.434 *P* < 0.001*
**Range**	50–100	0–50	
**Role emotional**			
**Mean ± SD**	90 ± 16.84	16.67 ± 25.42	*z* = −8.494 *P* < 0.001*
**Range**	33.33–100	0–100	
**Bodily pain**			
**Mean ± SD**	74.40 ± 13.25	56.70 ± 16.30	*t* = 5.958 *P* < 0.001*
**Range**	45–100	22.5–87.5	
**General health**			
**Mean ± SD**	66.90 ± 15.05	38.64 ± 10.44	*t* = 10.911 *P* < 0.001*
**Range**	40–100	1–60	
**Validity**			
**Range**	50	50	
**Social functioning**			
**Mean ± SD**	66.75 ± 17.06	39.05 ± 18.18	*z* = −6.911 *P* < 0.001*
**Range**	25–100	2.5–87.5	
**Mental health**			
**Mean ± SD**	48.08 ± 5.21	46.96 ± 4.56	*t* = 1.145 *P* = 0.255
**Range**	40–60	40–60	
**Physical health dimension**			
**Mean ± SD**	318.26 ± 70.94	158.52 ± 42.18	*t* = 13.686 *P* < 0.001*
**Range**	172–442	89–225	
**Mental health dimension**			
**Mean ± SD**	235.68 ± 23.22	151.22 ± 19.83	*t* = 19.563 *P* < 0.001*
**Range**	203–291	110–213	
**Physical mental aspect dimension**			
**Mean ± SD**	1384.85 ± 215.29	774.35 ± 119.36	*t* = 17.537 *P* < 0.001*
**Range**	940−1820	557–982	
**Total score**			
**Mean ± SD**	79.46 ± 11.95	44.05 ± 6.93	*t* = 18.126 *P* < 0.001*
**Range**	55–103.89	32.92–56.67	

Data are expressed as mean ± SD / median (range)

*t*, independent samples *t*-test; *z*, Mann-Whitney *U* test

*Statistically significant (*P* < 0.05)

## Discussion

This study is a randomized controlled trial of children aging 4–18 years with FC diagnosed by a general practitioner or pediatrician. The study group is consisted of 200 children receiving TRP plus pharmaceutical treatment, and a control group consisted of 200 children receiving pharmaceutical treatment only. Follow-up after 6 months, the outcome was compared according to the Rome III criteria to assess symptoms of FC and SF-36 form to assess QL. Adding TRP to pharmaceutical treatment of FC in children results in prominent improvement in the condition, there is a significant difference between the study and control group in all Rome criteria which assess symptoms of FC except rush to the bath-room to poop which showed non-significant difference, there is also a significant difference between the study and control group in all domains of the SF-36 questionnaire which assess QL except the mental health domain which showed non-significant difference.

The results of this study agreed with Silva et al. [38]. Patients aged 4 to 18 years old with FC according to the Rome III criteria were allocated to receive either physical therapy or drug intervention. In the physiotherapy group, a qualified physiotherapist used abdominal massage, isometric abdominal muscle, and diaphragmatic breathing exercises during 40-min sessions twice a week for 6 weeks, along with laxatives. Patients in the drug group only received laxatives. The findings after 6 weeks of interventions revealed that the physiotherapy group had more episodes of stool movements than the drug group. The incidence of fecal incontinence was similar between the groups, and the study concluded that combining isometric abdominal muscle training, breathing exercises, and abdominal massage raised defecation frequency after 6 weeks, suggesting that physiotherapy can be used as a complementary therapy for constipated children.

In addition, in 2017, research [39] was undertaken on children with dysfunctional voiding and persistent constipation who had failed primary care therapies. The children were divided into three groups in the prospective clinical investigation. All groups received education and behavioral changes. Furthermore, study group A got both interferential current stimulation and diaphragmatic breathing exercises, while study group B only got diaphragmatic breathing exercises. The therapy lasted 2 weeks in the clinic for all three groups. Only study group A experienced substantial improvement in defecation frequency and fecal incontinence following therapy. These youngsters saw considerable improvements in lower urinary tract symptoms and post-void residual pee. A bell-shaped uroflowmetry curve was seen in 73.3% of intervention group participants.

Interventions in physical therapy may be essential in raising the therapeutic outcome percentage. They consist of pelvic floor exercises and diaphragmatic breathing that teach children awareness, appropriate muscular function, and relaxation during defecation either with or without biofeedback [40]. A small Korean study [41] showed that in hospitalized children with developmental delays, abdominal meridian massage with aromatherapy oils reduced the frequency of medication and enema usage and increased the number of bowel movements and volume of stool. In a study conducted in 2014 [42], with daily abdominal massages for 6 weeks, 87.5% of children with physical limitations experienced symptomatic relief from constipation, 58% reduced their use of laxatives, and 41% increased their nutritional intake.

To assess the efficacy and safety of non-pharmacologic therapies for the treatment of children FC, a systematic review and meta-analysis was carried out in 2022 [43]. The study recommended that the strategies behind abdominal massage’s ability to reduce constipation are most likely a combination of local activation and relaxation, as well as activation of the parasympathetic nervous system. The findings from RCTs that were excluded from the meta-analyses recommended that some prebiotic and fiber blends, Chinese herbal medicine, and abdominal massage are promising therapies. Stretch receptors that can strengthen the gastro-colic response and cause intestinal and rectal contraction are activated when direct pressure is applied to the abdominal wall. This pressure alternatively compresses and releases portions of the digestive system, momentarily altering lumen size.

Combined both abdominal muscle and pelvic floor retraining is useful in the majority of children with dysfunctional voiding for curing urinary incontinence, constipation, nocturnal enuresis, and urinary tract infections [44]. The fact that the diaphragmatic breathing techniques were simple to teach to the children and helped them practice abdominal relaxation is impressive. The diaphragm travels caudally during diaphragmatic breathing inspiration, expanding the thoracic chamber and causing the anterior abdominal wall to protrude outward. It has been demonstrated that in healthy women, bulging lowers urethral pressure, which should make urinating and defecating easier [45].

Treatment for FC focuses on treating manifestations, particularly stomach discomfort and encopresis, which may contribute to avoidance of social events and decreased self-esteem. Overcoming this challenge is crucial in improving the QL [46]. Our study showed a significant difference between the intervention and control group in almost all domains of QL after 6 months of interventions; this is in accordance with the results of a study conducted in 2022 [46] and stated that regular effective treatment of FC in children results in a significant increase in all areas of life quality and its total scores. The study’s limitations included the fact that tests were not timed to account for the subjects’ hormone variations as they aged. In addition, more investigation is required to investigate if the efficacy of physiotherapy may be anticipated by patient variables or psychosocial situations associated with commencement.

## Conclusion

After treatment for 6 months, adding the telerehabilitation home program to pharmaceutical treatment of FC in children results in prominent improvement in symptoms of FC and QL.

### Supplementary Information

Supplementary Material 1: (PDF 279 kb)

Supplementary Material 2: (PDF 588 kb)

Supplementary Material 3: (PDF 1413 kb)

## Data Availability

No datasets were generated or analyzed during the current study.

## Abbreviations

FC: Functional constipation
GIT: Gastrointestinal tract
RCT: Randomized controlled trial
SF-36: Short form 36
TRP: Telerehabilitation home program
