# Supplementary material for: Effect of adding telerehabilitation home program to pharmaceutical treatment on the symptoms and the quality of life in children with functional constipation: a randomized controlled trial
Source: Eur J Pediatr. 2024 Jun 26;183(9):3943–58. doi: 10.1007/s00431-024-05639-8 (PMC11322404; doi:10.1007/s00431-024-05639-8)
Supplement: Supplementary file 1 — (PDF 279 kb) [file 431_2024_5639_MOESM1_ESM.pdf]

استبيان صحي

الجنس☐ ذكر

☐ انثى

العمر---------سنة

المؤهل العلمي: ابتدائي ☐

اعدادي ☐

ثانوي ☐

بكالوريوس ☐

ماجستير ☐

دكتوراه ☐

من فضلك، أجب على كل الأسئلة الموجودة في هذا الاستبيان. في حالة عدم وضوح أي سؤال، أرجو اختيار أقرب اجابة لمفهومك للسؤال.

١- بصورة عامة، كيف ترى حالتك الصحية؟

(اختر اجابة واحدة وضع علامة ✓ أمام الاجابة المناسبة)

☐ ممتازة

☐ جيدجدا

☐ جيدة

☐ لا بأس بها

☐ سيئة

٢- مقارنة بعام مضى، كيف تقيم حالتك الصحية الآن بصورة عامة؟

(اختر اجابة واحدة وضع علامة ✓ أمام الاجابة المناسبة)

☐ أفضل بكثير مما كانت عليه قبل عام

☐ أفضل نوعا ما من العام الماضي

☐ تقريبا على ما هي عليه

☐ أسوأ نوعا ما من العام الماضي

☐ أسوأ بكثير مما كانت عليه قبل عام

٣- تتعلق البنود التالية بأنشطة يمكن ان تقوم بها خلال يومك العادي.

في الوقت الحالي، الى اي مدى تقيدك حالتك الصحية:

(اختر اجابة واحدة وضع علامة ✓ تحت الاجابة المناسبة)

	نعم تقيدني كثيرا	نعم تقيدني قليلا	لا تقيدني اطلاقا
أ) من ممارسة الأنشطة الشاقة مثل: الجري، حمل الاشياء الثقيلة او مزاولة الأنشطة الرياضية المجهدة جدا؟	☐	☐	☐
ب) من ممارسة الأنشطة متوسطة الجهد، كتحريك الطاولة او التنظيف باستخدام المكنسة الكهربائية او تنظيف حديقة المنزل والعناية بها ؟	☐	☐	☐
ج) من حمل المشتريات من البقالة او السوق المركزي (السوبرماركت)؟	☐	☐	☐
د) من صعود الدرج لعدة ادوار؟	☐	☐	☐
هـ) من صعود الدرج لدور واحد فقط؟	☐	☐	☐
و) من الانحناء او الركوع او السجود ؟	☐	☐	☐
ز) من المشي لأكثر من كيلومتر ونصف؟	☐	☐	☐
ح) من المشي لمسافة نصف كيلومتر؟	☐	☐	☐
ط) من المشي لمسافة مئة متر؟	☐	☐	☐
ي) من الاستحمام او ارتداء الملابس بنفسك؟	☐	☐	☐

الصحة الجسمية

٤- تتعلق البنود التالية (أ ، ب ، ج ، د) بالمشاكل التي يمكن ان تواجهـــك خلال تأديتك لعملك او للأنشطة اليومية المعتادة نتيجة لحالتك الصحية الجسمية.

خلال الأسابيع الأربعة الماضية، هل تسببت حالتك الصحية الجسمية في:

(اختر اجابة واحدة وضع علامة ✓ تحت الاجابة المناسبة)

	نعم	لا
أ) **التقليل من الوقت** الذي تقضيه في العمل او اي انشطة اخرى؟	☐	☐
ب) **التقليل** مما تود **انجازه** من العمل أو أي أنشطة أخرى؟	☐	☐
ج) **تقييدك في أداء** نوع معين من الأعمال أو أي أنشطة أخرى؟	☐	☐
د) **أن تجد صعوبة في** تأدية العمل أو أي أنشطة أخرى؟ (على سبيل المثال، احتجت الى جهد اضافي لتأديتها)	☐	☐

الصحة النفسية

٥- تتعلق البنود التالية (أ ، ب ، ج) بالمشاكل التي يمكن ان تواجهك خلال تأديتك لعملك او الانشطة اليومية المعتادة كنتيجة لحالتك الصحية النفسية. (مثلا الشعور بالاكتئاب او القلق)

خلال الاسابيع الأربعة الماضية، هل تسببت حالتك الصحية النفسية في:

(اختر اجابة واحدة وضع علامة ✓ تحت الاجابة المناسبة)

	نعم	لا
أ) **التقليل من الوقت** الذي تقضيه في العمل او اي انشطة اخرى؟	☐	☐
ب) **التقليل** مما تود **انجازه** من العمل أو أي أنشطة أخرى؟	☐	☐
ج) **عدم انجاز** العمل او اي انشطة اخرى **بالحرص المعتاد**؟	☐	☐

الصحة الجسمية او النفسية

٦- **خلال الاسابيع الاربعة الماضية**، الى اي مدى تعارضت صحتك **الجسمية** او **النفسية** مع تأديتك لنشاطاتك الاجتماعية المعتادة مع عائلتك او اصدقائك او جيرانك او اي من المناسبات الاجتماعية الأخرى؟

(اختر اجابة واحدة وضع علامة ✓ أمام الاجابة المناسبة)

- ☐ لم يكن هناك أي تعارض اطلاقا
- ☐ كان هناك تعارض قليل
- ☐ كان هناك تعارض متوسط
- ☐ كان هناك تعارض كبير
- ☐ كان هناك تعارض كبير جدا

شدة الألم

٧- ما شدة **الألم الجسمي** الذي عانيت منه خلال **الاسابيع الاربعة الماضية**؟

(اختر اجابة واحدة وضع علامة ✓ أمام الاجابة المناسبة)

- ☐ لم يكن هناك أي ألم
- ☐ كان هناك ألم خفيف جدا
- ☐ كان هناك ألم خفيف
- ☐ كان هناك ألم متوسط
- ☐ كان هناك ألم شديد
- ☐ كان هناك ألم شديد جدا

٨- **خلال الاسابيع الاربعة الماضية**، الى اي مدى ادى **الألم الجسمي** الى التعارض مع تأديتك لأعمالك المعتادة (سواء داخل المنزل او خارجه)؟

(اختر اجابة واحدة وضع علامة ✓ أمام الاجابة المناسبة)

- ☐ لم يكن هناك أي تعارض
- ☐ كان هناك تعارض قليل جدا
- ☐ كان هناك تعارض متوسط
- ☐ كان هناك تعارض كبير
- ☐ كان هناك تعارض كبير جدا

٩- الأسئلة التالية تتعلق بكيفية شعورك وطبيعة سير الأمور معك **خلال الأسابيع الأربعة الماضية**، الرجاء اعطاء اجابة واحدة لكل سؤال بحيث تكون هذه الاجابة هي الأقرب الى الحالة التي كنت تشعر بها.

خلال الأسابيع الأربعة الماضية، كم من الوقت:

(اختر اجابة واحدة وضع علامة ✓ تحت الاجابة المناسبة)

	في كل الأوقات	في معظم الأوقات	في كثير من الأوقات	في بعض الأوقات	في قليل من الأوقات	لم اشعر في أي وقت من الأوقات
أ) شعرت بأنك ملئ بالحيوية والنشاط؟	☐	☐	☐	☐	☐	☐
ب) كنت شخصا عصبيا جدا؟	☐	☐	☐	☐	☐	☐
ج) شعرت بأنك في حالة اكتئاب الى درجة لم يمكن معها ادخال السرور اليك؟	☐	☐	☐	☐	☐	☐
د) شعرت بالهدوء والطمأنينة؟	☐	☐	☐	☐	☐	☐
هـ) كانت لديك طاقة كبيرة؟	☐	☐	☐	☐	☐	☐
و) شعرت بالاحباط واليأس؟	☐	☐	☐	☐	☐	☐
ز) شعرت بأنك منهك (استُنفِذت قُواك)؟	☐	☐	☐	☐	☐	☐
ح) شعرت بأنك شخص سعيد؟	☐	☐	☐	☐	☐	☐
ط) شعرت بأنك تعبان؟	☐	☐	☐	☐	☐	☐

١٠- **خلال الاسابيع الأربعة الماضية**، ما مقدار الوقت الذي تعارضت فيه **صحتك الجسمية** او **مشاكلك النفسية** مع **نشاطاتك الاجتماعية (مثل زيارة الأصدقاء والأقارب وغير ذلك)** ؟

(اختر اجابة واحدة وضع علامة ✓ أمام الاجابة المناسبة)

- ☐ كان التعارض في كل الأوقات
- ☐ كان التعارض في معظم الأوقات
- ☐ كان التعارض في بعض الأوقات
- ☐ كان التعارض في قليل من الأوقات
- ☐ لم يكن هنالك تعارض في أي وقت من الأوقات

١١- ما مدى **صحة** او **خطأ كل** من العبارات التالية (أ ، ب ، ج ، د) بالنسبة الى حالتك الصحية؟

(اختر اجابة واحدة وضع علامة ✓ تحت الاجابة المناسبة)

	صحيحة بلا شك	صحيحة غالبا	لا اعلم	خطأ غالبا	خطأ بلا شك
أ) يبدو أنني أصاب بالمرض أسهل من الآخرين.	☐	☐	☐	☐	☐
ب) حالتي الصحية مساوية لأي شخص أعرفه.	☐	☐	☐	☐	☐
ج) أتوقع أن تسوء حالتي الصحية.	☐	☐	☐	☐	☐
د) حالتي الصحية ممتازة.	☐	☐	☐	☐	☐

****** شكرا لتعاونكم ******
