# Supplementary material for: Effect of adding telerehabilitation home program to pharmaceutical treatment on the symptoms and the quality of life in children with functional constipation: a randomized controlled trial
Source: Eur J Pediatr. 2024 Jun 26;183(9):3943–58. doi: 10.1007/s00431-024-05639-8 (PMC11322404; doi:10.1007/s00431-024-05639-8)
Supplement: Supplementary file 2 — (PDF 588 kb) [file 431_2024_5639_MOESM2_ESM.pdf]

Appendix 2

Rome III Diagnostic Questionnaire for the Pediatric Functional GI Disorders

Section C. Bowel Movements ("Poop," "Stool," "Number 2")

This section asks about your bowel movements. There are many words for bowel movements, such as "poop," "stool," "BMs," and "going to the bathroom for number 2." Your family may use another special word when they talk about poops.

1. In the last 2 months, how often did you usually have poops?
   1. __ 2 times a week or less often
   2. __ 3 to 6 times a week
   3. __ Once a day
   4. __ 2 to 3 times a day
   5. __ More than 3 times a day

2. In the last 2 months, what was your poop usually like?
   1. ___ Very hard
   2. ___ Hard
   3. ___ Not too hard and not too soft
   4. ___ Very soft or mushy
   5. ___ Watery
   6. ___ It depends (my poops are not always the same)

   2a. If your poops were usually hard, for how long have they been hard?

   0. ___ Less than 1 month
   1. ___ 1 month
   2. ___ 2 months
   3. ___ 3 or more months

3. In the last 2 months, did it hurt when you had a poop?

   0. ___ No
   1. ___ Yes

Circle a number for your answer to each question below.<br><br>**In the last 2 months, how often**	0%<br>of the<br>time<br><br>Never	25%<br>of the<br>time<br><br>Once in<br>a while	50%<br>of the<br>time<br><br>Sometimes	[illegible]5%<br>of the<br>time<br><br>Most of<br>the time	100%<br>of the<br>time<br><br>Always
4. Did you have to rush to the bath-room to poop?	0	1	2	3	4
5. Did you have to strain (push hard) to make a poop come out?	0	1	2	3	4
6. Did you pass mucus or phlegm (white, yellowish, stringy, or slimy material) during a poop?	0	1	2	3	4
7. Did you have a feeling of not being finished after a poop (like there was more that wouldn't come out)?	0	1	2	3	4

8. In the last 2 months, did you have a poop that was so big that it clogged the toilet?
   0. __ No
   1. __ Yes

9. Some children hold in their poop even when there is a toilet they could use. They may do this by stiffening their bodies or crossing their legs. In the last 2 months, while at home, how often did you try to hold in a poop?
   0. __ Never
   1. __ 1 to 3 times a month
   2. __ Once a week
   3. __ Several times a week
   4. __ Every day

10. Did a doctor or nurse ever examine you and say that you had a huge poop inside?
   0. __ No
   1. __ Yes

SCORING INSTRUCTIONS FOR PARENT-REPORT FORM AND CHILD/ADOLESCENT SELF-REPORT FORM

11. In the last 2 months, how often was your underwear stained or soiled with poop?

0. ____ Never. *If never, please go to Section D.*

1.____ Less than once a month

2.____ 1 to 3 times a month

3.____ Once a week

4.____ Several times a week

5.____ Every day

[illegible]*a.* When you stained or soiled underwear, how much was it stained or soiled?

1. __ Underwear was stained (no poop)

2. __ Small amount of poop in underwear (less than a whole poop)

3. __ Large amount of poop in underwear (a whole poop)

[illegible]*b.* For how long have you stained or soiled your underwear?

1. __ 1 month or less

2. __ 2 months

3. __ 3 months

4. __ 4 to 11 months

5. __ 1 year or longer
