# Supplementary material for: Effect of adding telerehabilitation home program to pharmaceutical treatment on the symptoms and the quality of life in children with functional constipation: a randomized controlled trial
Source: Eur J Pediatr. 2024 Jun 26;183(9):3943–58. doi: 10.1007/s00431-024-05639-8 (PMC11322404; doi:10.1007/s00431-024-05639-8)
Supplement: Supplementary file 3 — (PDF 1413 kb) [file 431_2024_5639_MOESM3_ESM.pdf]

SPRINGER NATURE

Change of authorship request form - Journals (pre-acceptance)

Important information. Please read.

- This form should be used by authors to request any change in authorship (adding/deleting authors) including changes in corresponding authors. This form should not be used for name changes. Please fully complete all sections. Use black ink and block capitals and provide each author's full name with the given name first followed by the family name.

- By signing this declaration, all authors guarantee that the order of the authors are in accordance with their scientific contribution, if applicable as different conventions apply per discipline, and that only authors have been added who made a meaningful contribution to the work.

- Please note, in author collaborations where there is formal agreement for representing the collaboration, it is sufficient for the representative or legal guarantor (usually the corresponding author) to complete and sign the Authorship Change Form on behalf of all authors, **next to the added/removed author(s)**. **(Complete Section 3, followed by Section 6.)**
  In author collaborations where there is no formal agreement for representing the collaboration and **there are more than 10 authors**, one may sign for all, provided the signer appends correspondence that attests that each of the authors have agreed to the change **and the added/removed authors sign the form**. **(Complete Section 3, followed by Section 6.)**

- Please note, we cannot investigate or mediate any authorship disputes. If you are unable to obtain agreement from all authors (including those who you wish to be removed) you must refer the matter to your institution(s) for investigation. Please inform us if you need to do this.

- If you are not able to return a fully completed form within **30 days** of the date that it was sent to the author requesting the change, we may have to withdraw your manuscript. We cannot publish manuscripts where authorship has not been agreed by all authors (including those who have been removed).

- Incomplete forms will be rejected.

- Please return/upload this form, fully completed, to the Journals Editorial Office. The Journal and/or Publisher will consider the information you have provided to decide whether to approve the proposed change in authorship. We may decide to contact your institution for more information or undertake a further investigation, if appropriate, before making a final decision.

SPRINGER NATURE

Change of authorship request form - Journals (pre-acceptance)

Section 1: Please provide the current title of manuscript

Title: Effect of Adding Telerehabilitation Home Program to Pharmaceutical Treatment on the symptoms and the Quality of life in Children with Functional Constipation: A Randomized Controlled Trial

Manuscript ID no.: 96049e3d-ff32-467d-be4c-567a1774786b v2.0

Section 2: Please provide the previous authorship, in the order shown on the manuscript before the changes were introduced. Please indicate the corresponding author by adding (CA) behind the name.

	First name(s)	Family name	ORCID or SCOPUS id, if available
1st author	Amir	Soliman	
2nd author	Safy. E.	Abo-Ali	
3rd author	Amel. E.	Abdel Karim	
4th author	Sara. A.	Elsamahy	
5th author	Judy	Hasan	
6th author	Badr Al-Amir	Hassan	
7th author	Amira H.	Mohammed	https://orcid.org/0000-0002-6018-9138

Please use an additional sheet if there are more than 10 authors.

Section 3: Please provide a justification for change. Please use this section to explain your reasons for changing the authorship of your manuscript, e.g. what necessitated the change in authorship? Please refer to the (journal) policy pages for more information about authorship. Please explain why omitted authors were not originally included and/or why authors were removed on the submitted manuscript.

We refer to the (journal) policy pages for more information about authorship. We did not add and/or delete authors at revision stage, we only reorder their arrangement in the title and affiliation page.

SPRINGER NATURE ---

Springer Nature is one of the world's leading global research, educational and professional publishers, created in May 2015 through the combination of Nature Publishing Group, Palgrave Macmillan, Macmillan Education and Springer Science+Business Media.

Change of authorship request form - Journals (pre-acceptance)

Section 4: Proposed new authorship. Please provide your new authorship list in the order you would like it to appear on the manuscript. Please indicate the corresponding author by adding (CA) behind the name. <u>If the Corresponding Author has changed, please indicate the reason under section 3</u>.

	First name(s)	Family name (this name will appear in full on the final publication and will be searchable in various abstract and indexing databases)	Affiliated institute	E-mail address
1st author	Amir	Soliman	Department of Public Health and Community Medicine, Faculty of Medicine, Delta University for Science and Technology, Gamasa, Egypt.	amir.soliman@deltauniv.edu.eg, amirsoliman0004@gmail.com
2nd author	Safy. E.	Abo-Ali	Department of Physical Therapy for Pediatrics, Faculty of physical therapy, October University for Modern Sciences and Arts, Egypt.	safy2001@yahoo.com
3rd author	Amel. E.	Abdel Karim	Department of Physical Therapy for Pediatrics, Misr University for Science and Technology, Giza, Egypt.	amel64@hotmail.com.
4th author	Sara. A.	Elsamahy	Basic Science Department, Faculty of Physical Therapy, Misr University for Science and Technology, Egypt.	Sara.samahi@must.edu.eg
5th author	Judy	Hasan	Student in STEM school, Al Dakahleya, Egypt.	Judyamer267@gmail.com

6th author	Badr Al-Amir	Hassan	Department of physical therapy for internal medicine and geriatrics, Faculty of Physical Therapy, Delta University for Science and Technology, Gamasa, Egypt.	badr.alamer@deltauniv.edu.eg
7th author	Amira H.	Mohammed **(CA)**	Department of Physical Therapy for Pediatrics, Faculty of Physical Therapy, Delta University for Science and Technology, Gamasa, Egypt.	amira_hussin77@yahoo.com, amira.hussien@deltauniv.edu.eg

Please use an additional sheet if there are more than 10 authors.

Section 5: Author contribution, Acknowledgement and Disclosures. Please use this section to provide a new disclosure statement and, if appropriate, acknowledge any contributors who have been removed as authors and ensure you state what contribution any new authors made (if applicable per the journal or book (series) policy). Please ensure these are updated in your manuscript - after approval of the change(s) - as our production department will not transfer the information in this form to your manuscript.

We did not add and/or delete authors at revision stage, we only reorder their arrangement in the title and affiliation page.

New acknowledgements:

New Disclosures (financial and non-financial interests, funding):

New Author Contributions statement (if applicable per the journal policy):

State 'Not applicable' if there are no new authors.

SPRINGER NATURE **Change of authorship request form - Journals (pre-acceptance)**

Section 6: Declaration of agreement. *All* authors, unchanged, new and removed *must* sign this declaration.
(NB: Please print the form, (docu)-sign and return/upload a scanned copy. Please note that signatures that have been inserted as an image file are acceptable as long as it is handwritten. Typed names in the signature box are unacceptable.) *** Please delete as appropriate. Delete all of the bold if you were on the original authorship list and are remaining as an author.**

	First name	Family name		Signature	Date
1st author	Amir	Soliman	I agree to the proposed new authorship shown in section 4 **/and the addition/removal*of my name to the authorship list** /and the proposed change in corresponding author	Amir Soliman	6-4-2024
2nd author	Safy. E.	Abo-Ali	I agree to the proposed new authorship shown in section 4 **/and the addition/removal*of my name to the authorship list** /and the proposed change in corresponding author	Safy Abo Ali	5-4-2024
3rd author	Amel. E.	Abdel Karim	I agree to the proposed new authorship shown in section 4 **/and the addition/removal*of my name to the authorship list** /and the proposed change in corresponding author	Amel Abdel Kariem	5-4-2024
4th author	Sara. A.	Elsamahy	I agree to the proposed new authorship shown in section 4 **/and the addition/removal*of my name to the authorship list** /and the proposed change in corresponding author	Sara	5-4-2024
5th author	Judy	Hasan	I agree to the proposed new authorship shown in section 4 **/and the addition/removal*of my name to the authorship list** /and the proposed change in corresponding author	Judy Hasan	5-4-2024
6th author	Badr Al-Amir	Hassan	I agree to the proposed new authorship shown in section 4 **/and the addition/removal*of my name to the authorship list** /and the proposed change in corresponding author	Badr Hassan	6-4-2024
7th author	Amira H.	Mohammed **(CA)**	I agree to the proposed new authorship shown in section 4 **/and the addition/removal*of my name to the authorship list** /and the proposed change in corresponding author	Amira. Hussin	6-4-2024

SPRINGER NATURE —
Springer Nature is one of the world's leading global research, educational and professional publishers, created in May 2015 through the combination of Nature Publishing Group, Palgrave Macmillan, Macmillan Education and Springer Science+Business Media.

Change of authorship request form - Journals (pre-acceptance)

Please use an additional sheet if there are more than 10 authors.

In case of author collaborations with formal agreement:

	Name of consortium/consortia	First name	Family name		Signature	Date
Representative/legal guarantor				I agree to the proposed new authorship shown in section 4 /**and the addition/removal*of my name to the authorship list** /and the proposed change in corresponding author		

Both added/removed authors should complete the information in the first table under Section 6.

---- End of form ----
